# Supplementary material for: SARS-CoV-2 VOC type and biological sex affect molnupiravir efficacy in severe COVID-19 dwarf hamster model
Source: Nat Commun. 2022 Jul 29;13:4416. doi: 10.1038/s41467-022-32045-1 (PMC9338273; doi:10.1038/s41467-022-32045-1)
Supplement: Supplementary file 1 — Supplementary Information [file 41467_2022_32045_MOESM1_ESM.pdf]

SARS-CoV-2 VOC type and biological sex affect molnupiravir efficacy in severe COVID-19 dwarf hamster model

Carolin M Lieber[1#], Robert M Cox[1#], Julien Sourimant[1], Josef D Wolf[1], Kate Juergens[2], Quynh Phung[2], Manohar T Saindane[3], Megan K Smith[3], Zachary M Sticher[3], Alexander A Kalykhalov[3], Michael G Natchus[3], George R Painter[3], Kaori Sakamoto[4], Alexander L. Greninger[2], Richard K Plemper[1*]

[1]Center Translational Antiviral Research, Institute for Biomedical Sciences, Georgia State University, Atlanta, 30303 GA

[2]Virology Division, Department of Laboratory Medicine, University of Washington, Seattle, 98185 WA

[3]Emory Institute for Drug Development, Emory University, Atlanta, GA 30329

[4]Department of Pathology, College of Veterinary Medicine, University of Georgia, Athens, GA 30602

[#]these authors contributed equally to this work

Supplementary Information

Supplementary Table 1. Whole genome sequencing summary of VOC reisolated from dwarf hamsters.

Supplementary Table 2. Selected single oral dose PK parameters of molnupiravir in dwarf hamsters.

Supplementary Fig. 1. Transepithelial electrical resistance measurements (TEER).

Supplementary Fig. 2. Confocal images of SARS-CoV-2 infection in HAEs with stained goblet cells.

Supplementary Fig. 3. Confocal images of SARS-CoV-2 infection in HAEs with stained ciliated cells.

Supplementary Fig. 4. Infection of ferrets with VOC beta.

Supplementary Fig. 5. Viral RNA and upper respiratory tract viral titers from VOC delta infected ferrets.

Supplementary Fig. 6. Viral RNA and upper respiratory tract viral titers from VOC alpha, gamma, and omicron infected ferrets.

Supplementary Fig. 7: Lung pathology in Roborovski dwarf hamsters infected with different VOC.

Supplementary Fig. 8. Lung pathology in dwarf hamsters infected with VOC gamma or delta.

Supplementary Fig. 9. Clinical signs during treatment with molnupiravir.

Supplementary Fig. 10. SARS-CoV-2 RNA copies in dwarf hamsters infected with different VOC.

Supplementary Fig. 11. Effect of biological sex on survival of vehicle treated dwarf hamsters.

Supplementary Fig. 12. Lungs of vehicle treated infected dwarf hamsters.

Supplementary Fig. 13. Lungs of molnupiravir treated infected dwarf hamsters.

Supplementary Fig. 14. Histopathology of dwarf hamster lungs 3 days after infection.

Supplementary Fig. 15. Histopathology of dwarf hamster lungs 14 days after infection.

	vehicle		molnupiravir	
VOC	nsp6	spike	nsp6	spike
delta	0/4	N950D (2/4)	0/4	N950D (2/5)
		D142G (4/4)		D142G (4/5)
		A653V (0/4)		A653V (1/5)
		V341I (0/4)		V341I (1/5)
gamma	K8N (0/9)	N20T (0/9)	K8N (1/9)	N20T (1/9)
	T77A (0/9	E132K (0/9)	T77A (1/9)	E132K (1/9)
	V117L (0/9)	G268C (0/9)	V117L (1/9)	G268C (1/9)
	V181F (9/9)	S375P (0/9)	V181F (8/9)	S375P (1/9)
	G255R (0/9)	T417K (0/9)	G255R (1/9)	T417K (1/9)
	F257L (4/9)	L452R (0/9)	F257L (3/9)	L452R (1/9)
	A846T (1/9)	T478K (0/9)	A846T (1/9)	T478K (0/9)
		K484E (1/9)		K484E (1/9)
		Y501N (0/9)		Y501N (1/9)
		Y655H (0/9)		Y655H (1/9)
		A903D (0/9)		A903D (1/9)
		Y904F (0/9)		Y904F (1/9)
		G932C (0/9)		G932C (1/9)
		K933N (0/9)		K933N (1/9)
		K933I (0/9)		K933I (1/9)
		D936Y (0/9)		D936Y (1/9)
		V1189I (1/9)		V1189I (0/9)
		Y1206D (0/9)		Y1206D (1/9)
omicron	L260F (11/11)	L369F (0/11)	L260F (16/16)	L369F (1/16)
		L369P (0/11)		L369P (1/16)
		P371S (0/11)		P371S (1/16)
		F373S (0/11)		F373S (1/16)
		N415K (1/11)		N415K (3/16)
		K438N (2/11)		K438N (3/16)
		R496Q (1/11)		R496Q (0/16)
		Y499N (1/11)		Y499N (0/16)
		H503Y (1/11)		H503Y (0/16)

Supplementary Table 1. Whole genome sequencing summary of VOC reisolated from dwarf hamsters. Animals were infected and treated as in Fig. 4a. Shown are mutations in nsp6 and spike that were absent from virus inoculum and reached a relative allele frequency >20% with a depth of ≥10 reads. Numbers in parenthesis specify individual animals harboring viruses with the mutation per number of animals with readable sequencing results in this group. Mutations in dark grey shading reached a relative frequency of >75% in all animals of the group. Whole genome sequencing data for all animals are shown in Supplementary Datasets 1-3.

biological sex	t_{max} [h]	C_{max} [mol/ml]	AUC_{inf} [h*nmol/ml]	AUC_{inf}/dose [h*kg*nmol/ml/mmol]	$t_{1/2}$ [h]
male	0.5	0.876	1.42	1.88	1.23
female	0.5	1.06	1.57	2.06	2.45
combined	0.5	0.967	1.45	1.90	1.62

Supplementary Table 2. Selected single oral dose PK parameters of molnupiravir in dwarf hamsters. Shown are calculations based on NHC plasma concentrations. Samples were analyzed by a qualified LC-MS/MS method, calculations with WinNonlin (n=4 per time point assessed).

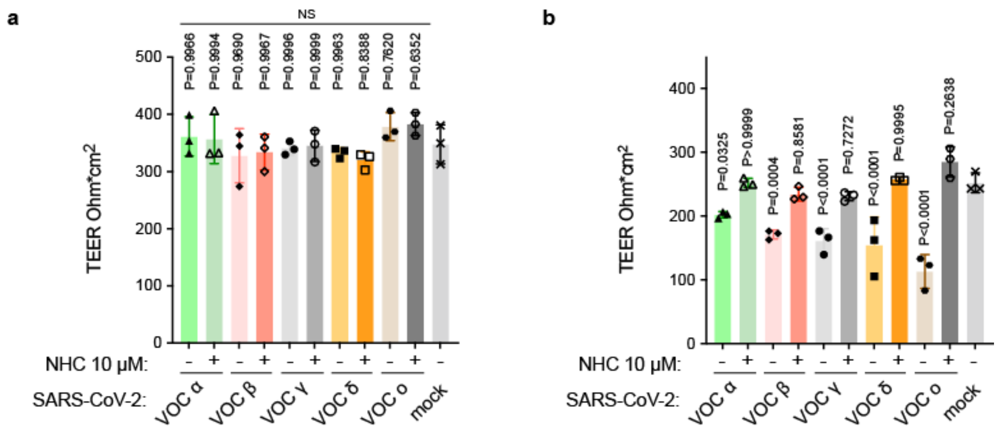

Supplementary Fig. 1. Transepithelial electrical resistance measurements (TEER). HAE cultures were mock infected (× symbols) or infected with VOC alpha (triangles), beta (diamonds), gamma (circles), delta (squares), or omicron (hexagons) in the presence of 10 µM NHC (open symbols) or vehicle (0.1% DMSO; closed symbols). **a**, TEER measurements of HAE cultures taken at the time of infection with different SARS-CoV-2 VOC; n=3 each. **b**, TEER measurements of HAE cultures taken 3 days after infection with different SARS-CoV-2 VOC; n=3 each. Symbols in (a-b) represent independent biological repeats (independent transwells). Color coding of VOC as specified is consistent across (a) and (b). P values are shown; NS, not significant. 1-way ANOVA with Dunnett's post hoc multiple comparison tests (a-b). Bar graphs show the group mean ± SD. Source data are provided as a Source Data file.

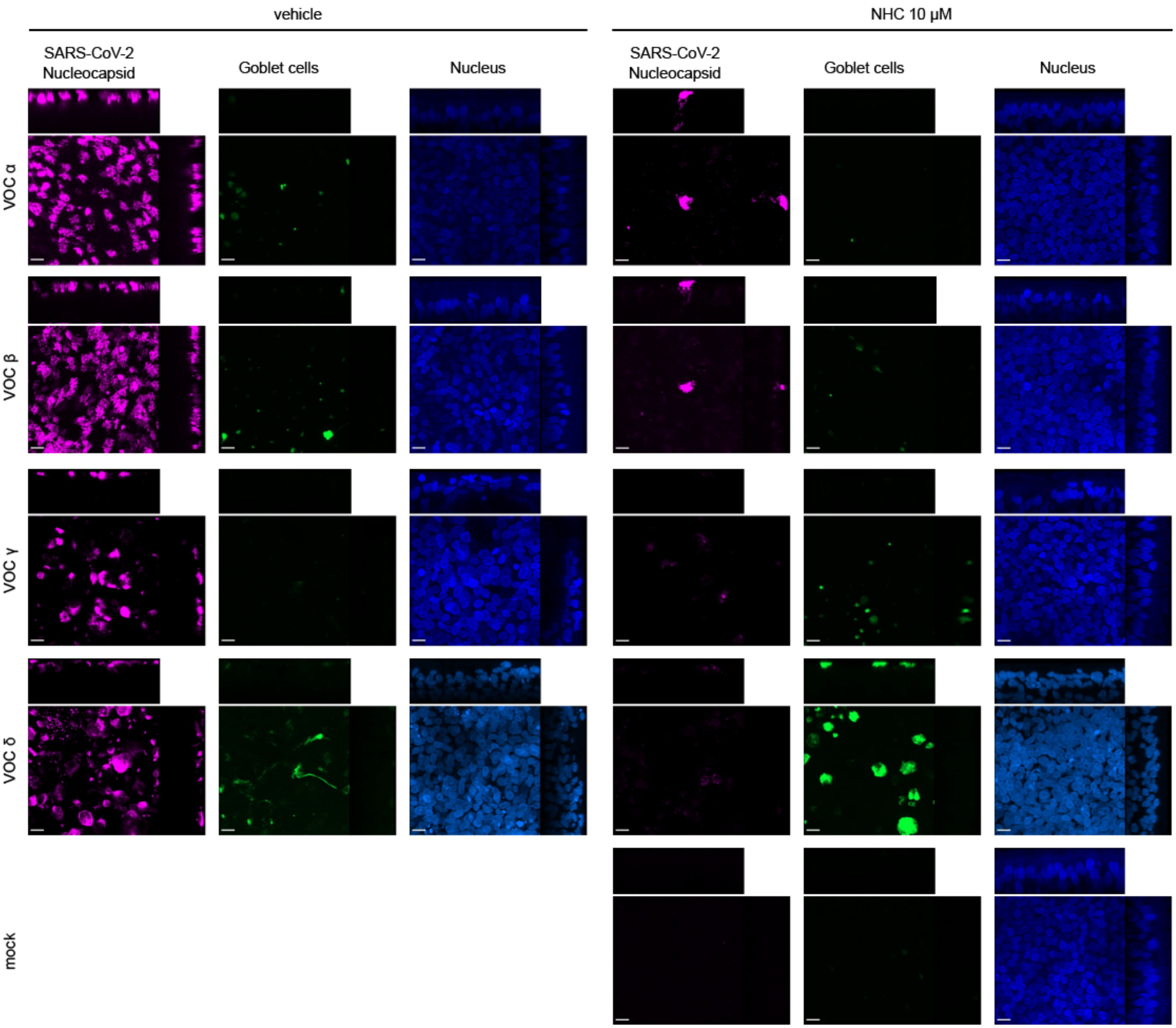

Supplementary Fig. 2. Confocal images of SARS-CoV-2 infection in HAEs with stained goblet cells. Images of HAE cultures infected with VOC. Organoids were apically infected with the specified VOC in the presence of basolateral vehicle (0.1% DMSO; left) or 10 µM NHC (right). Images were acquired on day 3 post infection. Images of HAE infected cultures in the presence and absence of NHC showing SARS-CoV-2 nucleocapsid (magenta), goblet cells (green) and nuclei (DAPI, blue). All images were acquired at 63× magnification. Per condition and viral target, two independent transwells were processed, one stained for SARS-CoV-2 nucleocapsid and one for SARS-CoV-2 spike (Supplementary Fig. 3); representative fields of view are shown. Scale bar represents 10 µM.

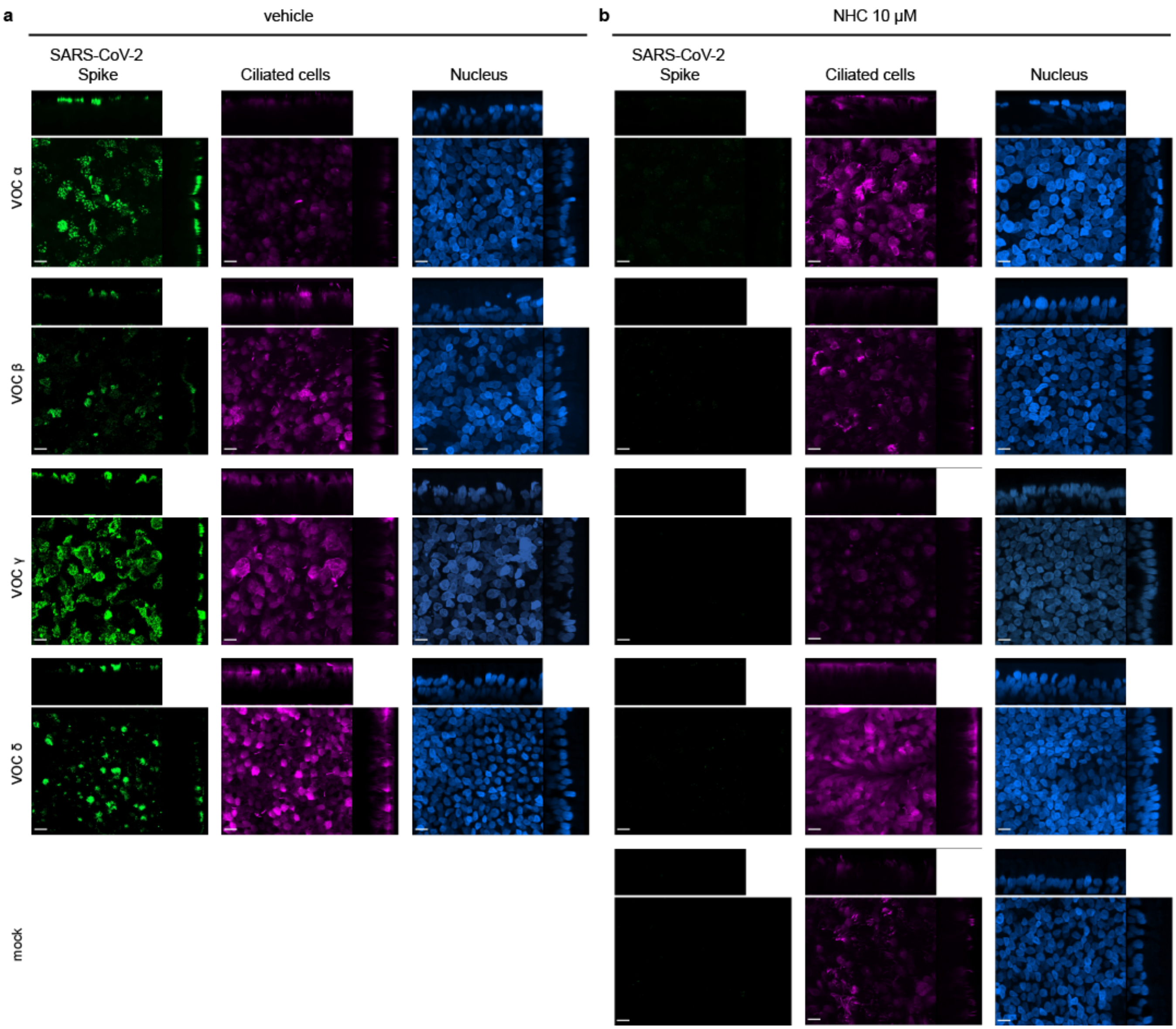

Supplementary Fig. 3. Confocal images of SARS-CoV-2 infection in HAEs with stained ciliated cells. Images of HAE cultures infected with VOC. Organoids were apically infected with the specified VOC in the presence of basolateral vehicle (0.1% DMSO, left) or 10 µM NHC (right). Images were acquired on day 3 post infection. Images of HAE infected cultures in the presence and absence of NHC showing SARS-CoV-2 Spike (green), ciliated cells (magenta), and nuclei (DAPI, blue). All images were acquired at 63× magnification. Per condition and viral target, two independent transwells were processed, one stained for SARS-CoV-2 spike and one for SARS-CoV-2 nucleocapsid (Supplementary Fig. 2); representative fields of view are shown. Scale bar represents 10 µM.

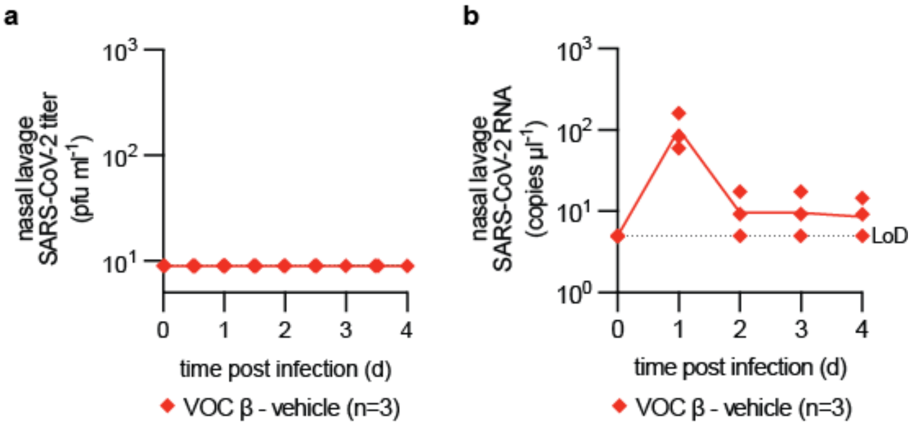

Supplementary Fig. 4. Infection of ferrets with VOC beta. **a**, SARS-CoV-2 infectious virus present in nasal lavages from ferrets infected with VOC beta and treated with vehicle. **b**, SARS-CoV-2 RNA copies present in nasal lavages from ferrets infected with VOC beta and treated with vehicle Infectious titers of SARS-CoV-2 in nasal turbinates harvested four days after infection. LoD limit of detection. Lines connect group medians. Symbols represent independent biological repeats (individual animals). Source data are provided as a Source Data file.

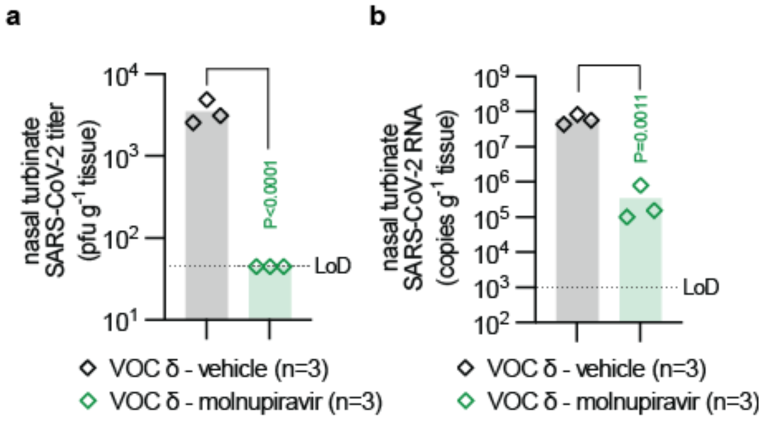

Supplementary Fig. 5. Viral RNA and upper respiratory tract viral titers from VOC delta infected ferrets. **a**, Infectious titers of SARS-CoV-2 in nasal turbinates harvested 4 days after infection. **b**, SARS-CoV-2 RNA copies present in nasal lavages from ferrets infected with VOC delta and treated with vehicle or molnupiravir. LoD limit of detection. Two-tailed t-test (a-b); P values are shown. Symbols represent independent biological repeats (individual animals). Bar graphs show the group mean. Source data are provided as a Source Data file.

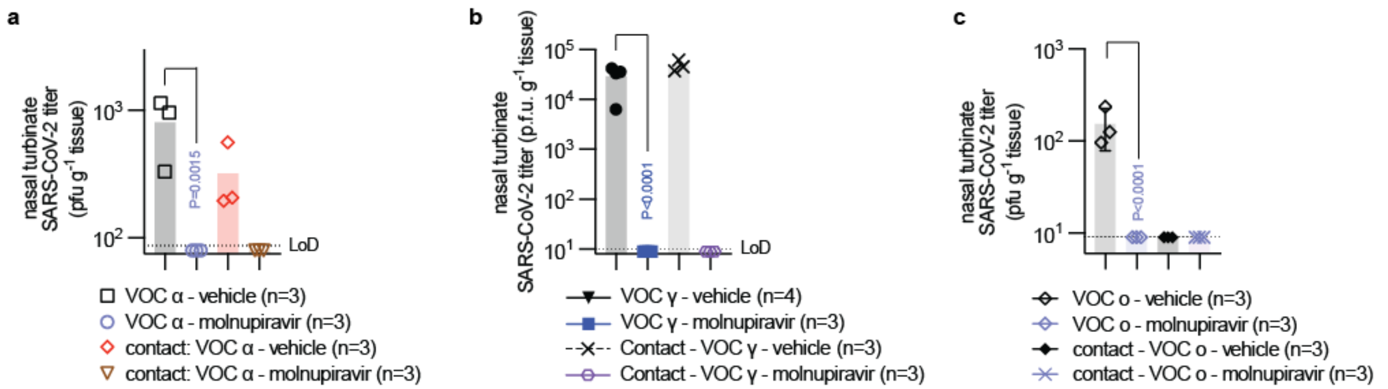

Supplementary Fig. 6. Viral RNA and upper respiratory tract viral titers from VOC alpha, gamma, and omicron infected ferrets. **a**, Infectious titers of SARS-CoV-2 VOC alpha in nasal turbinates harvested 4 days after infection of source ferrets treated with vehicle (black squares) or molnupiravir (blue circles) and of untreated vehicle (red diamonds) or molnupiravir (brown triangles) contact ferrets. **b**, Infectious titers of SARS-CoV-2 VOC gamma in nasal turbinates harvested 4 days after infection of source ferrets treated with vehicle (black triangles) or molnupiravir (blue squares) and of untreated vehicle (brown × symbols) or molnupiravir (purple hexagons) contact ferrets. **c**, Infectious titers of SARS-CoV-2 VOC gamma in nasal turbinates harvested 4 days after infection of source ferrets treated with vehicle (black diamonds) or molnupiravir (blue diamonds) and of untreated vehicle (black diamonds) or molnupiravir (blue × symbols) contact ferrets. LoD limit of detection. 1-way ANOVA with Tukey's (a-c) post hoc multiple comparison tests; P values are shown. Symbols represent independent biological repeats (individual animals). Bar graphs show the group mean. Error bars represent ± SD. Source data are provided as a Source Data file.

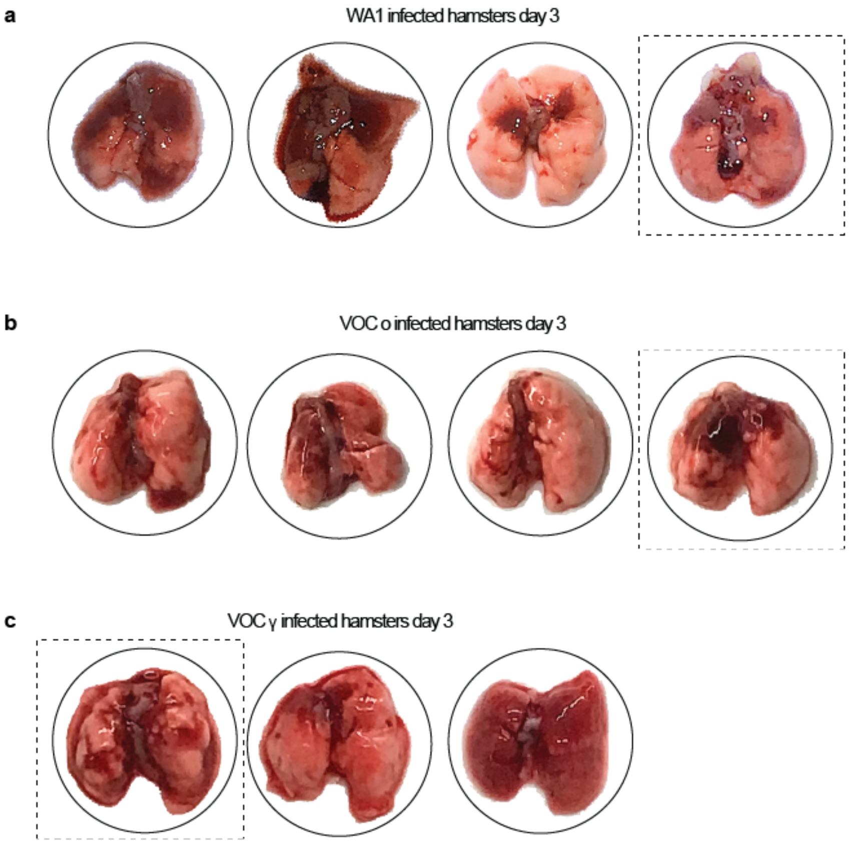

Supplementary Fig. 7. Lung pathology in Roborovski dwarf hamsters infected with different VOC. **a-c,** Lungs harvested from dwarf hamsters infected with 1×10^5 pfu of WA1 (a), VOC omicron (b), and VOC gamma (c) displayed acute lung injury and visible macroscopic lesions. Images boxed out (dashed boxes) represent lungs shown in Fig. 3f.

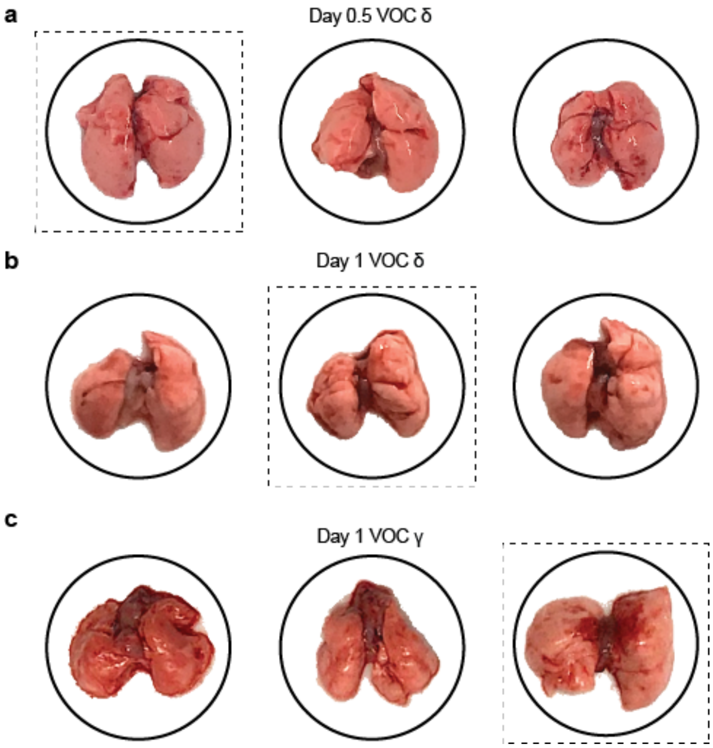

Supplementary Fig. 8. Lung pathology in dwarf hamsters infected with VOC gamma or delta. **a-b**, Images of lungs from hamsters inoculated with 1×10^4 pfu of VOC delta 0.5 days (a) and 1 day (b) after infection. **c**, Images of lungs from hamsters inoculated with 1×10^4 pfu of VOC gamma 1 day after infection. Images boxed out (dashed boxes) represent lungs shown in Fig. 3i.

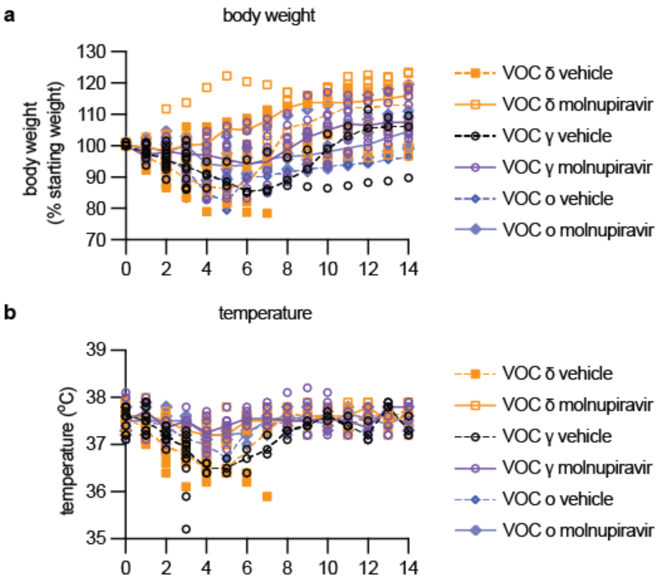

Supplementary Fig. 9. Clinical signs during treatment with molnupiravir. Roborovski dwarf hamsters infected with 1×10^4 pfu of VOC gamma, VOC delta, or VOC omicron and treated with molnupiravir or vehicle were infected and monitored for clinical signs for 14 days. **a-b**, Body weight (a) and temperature (b) were measured once daily. Symbols represent independent biological repeats (individual animals). Source data are provided as a Source Data file.

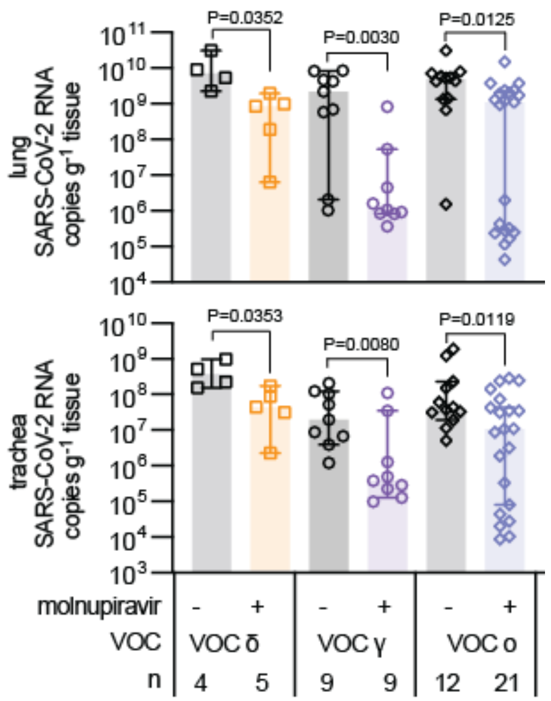

Supplementary Fig. 10. SARS-CoV-2 RNA copies in dwarf hamsters infected with different VOC. RNA copies were determined in dwarf hamsters infected with 1×10^4 pfu of VOC gamma, VOC delta, or VOC omicron treated with molnupiravir or vehicle. SARS-CoV-2 RNA copies present in lungs (top) and trachea (bottom). Symbols represent independent biological repeats (n, individual animals), columns show group medians and error bars represent the 95% confidence intervals. Significance was determined using unpaired two-tailed t-tests; P values are shown. Source data are provided as a Source Data file.

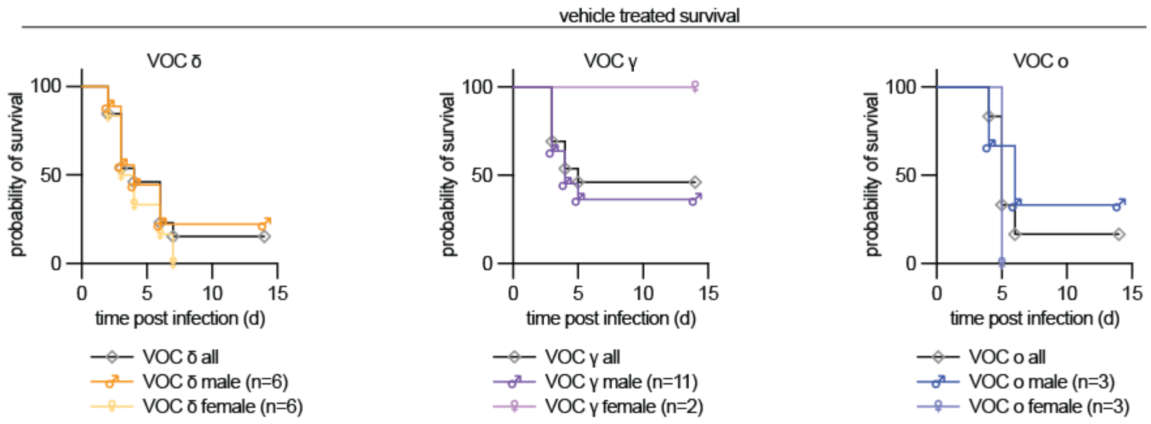

Supplementary Fig. 11. Effect of biological sex on survival of vehicle treated dwarf hamsters. Animals were infected with 1×10^4 pfu of VOC gamma, VOC delta, or VOC omicron. Survival curves for females (♀ symbol) and males (♂ symbol) are shown. Source data are provided as a Source Data file.

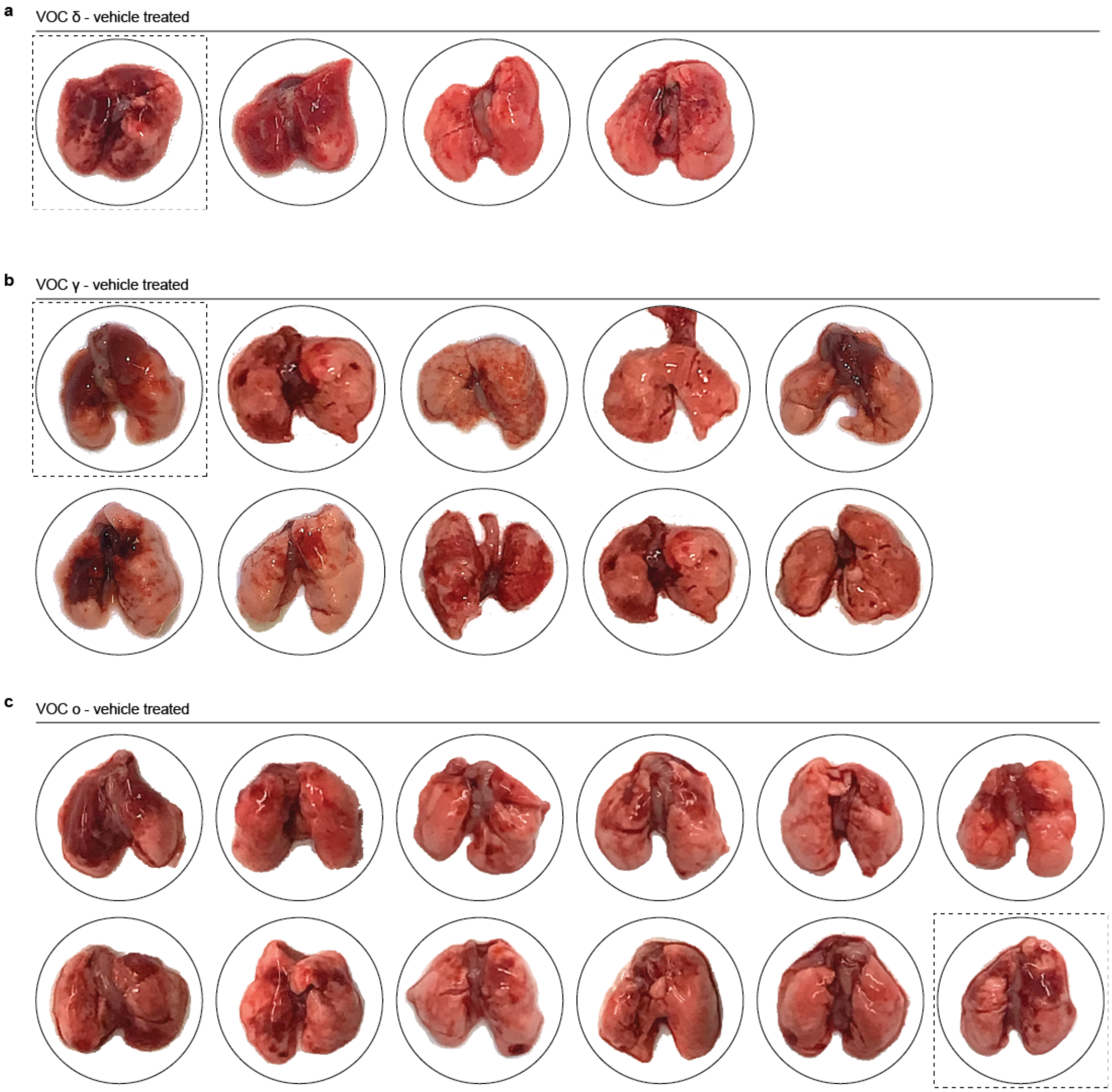

Supplementary Fig. 12. Lungs of vehicle treated infected dwarf hamsters. **a-c,** Images of lungs from hamsters mock infected or inoculated with 1×10^4 pfu of VOC delta (a), gamma (b), or omicron (c), treated with vehicle, and harvested 3 days after infection. Lungs boxed out (dashed boxes) shown in Fig. 6a.

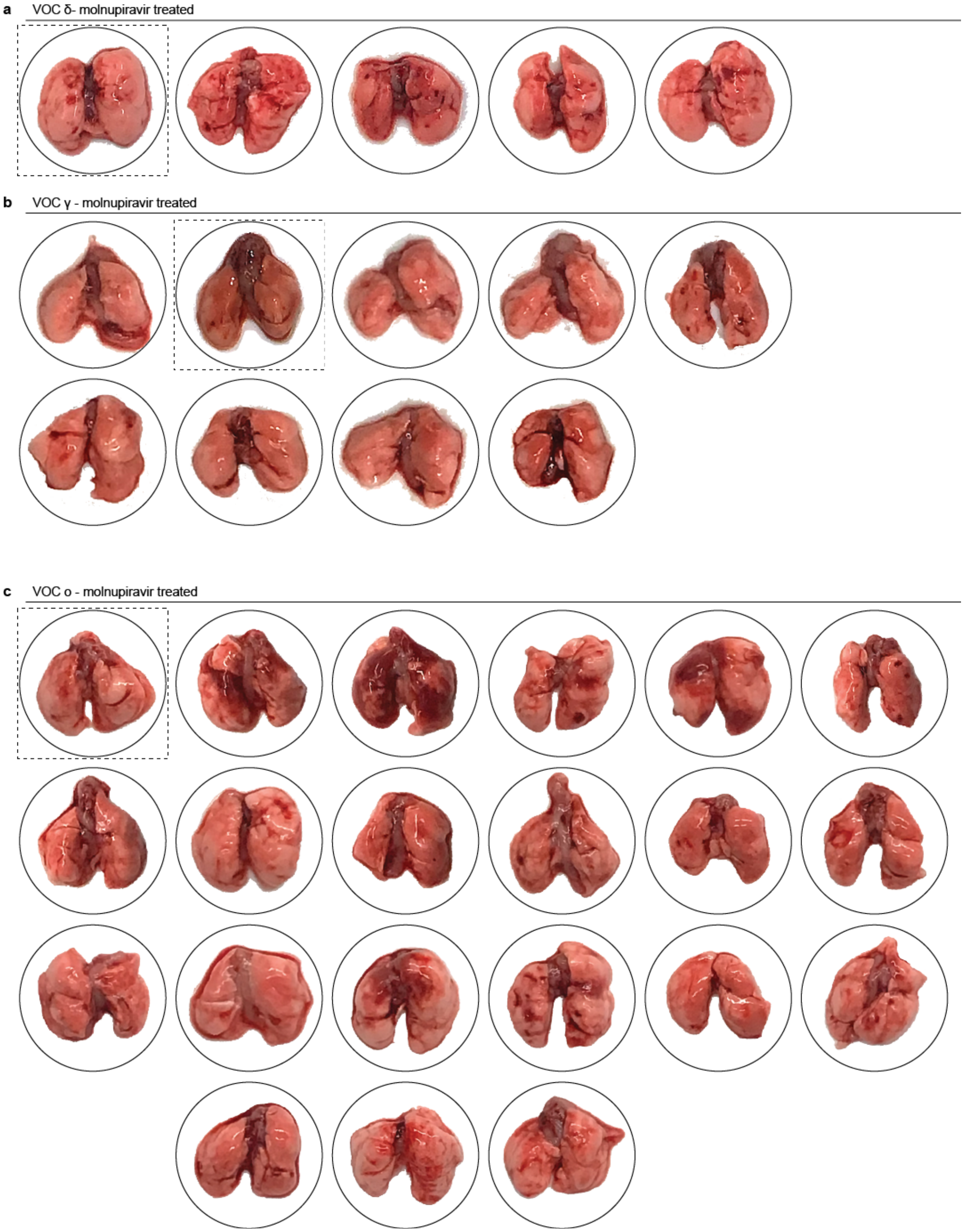

Supplementary Fig. 13. Lungs of molnupiravir treated infected dwarf hamsters. **a-c,** Images of lungs from hamsters mock infected or inoculated with 1×10^4 pfu of VOC delta (a), gamma (b), or omicron (c), treated with molnupiravir, and harvested 3 days after infection. Lungs boxed out (dashed boxes) shown in Fig. 6a.

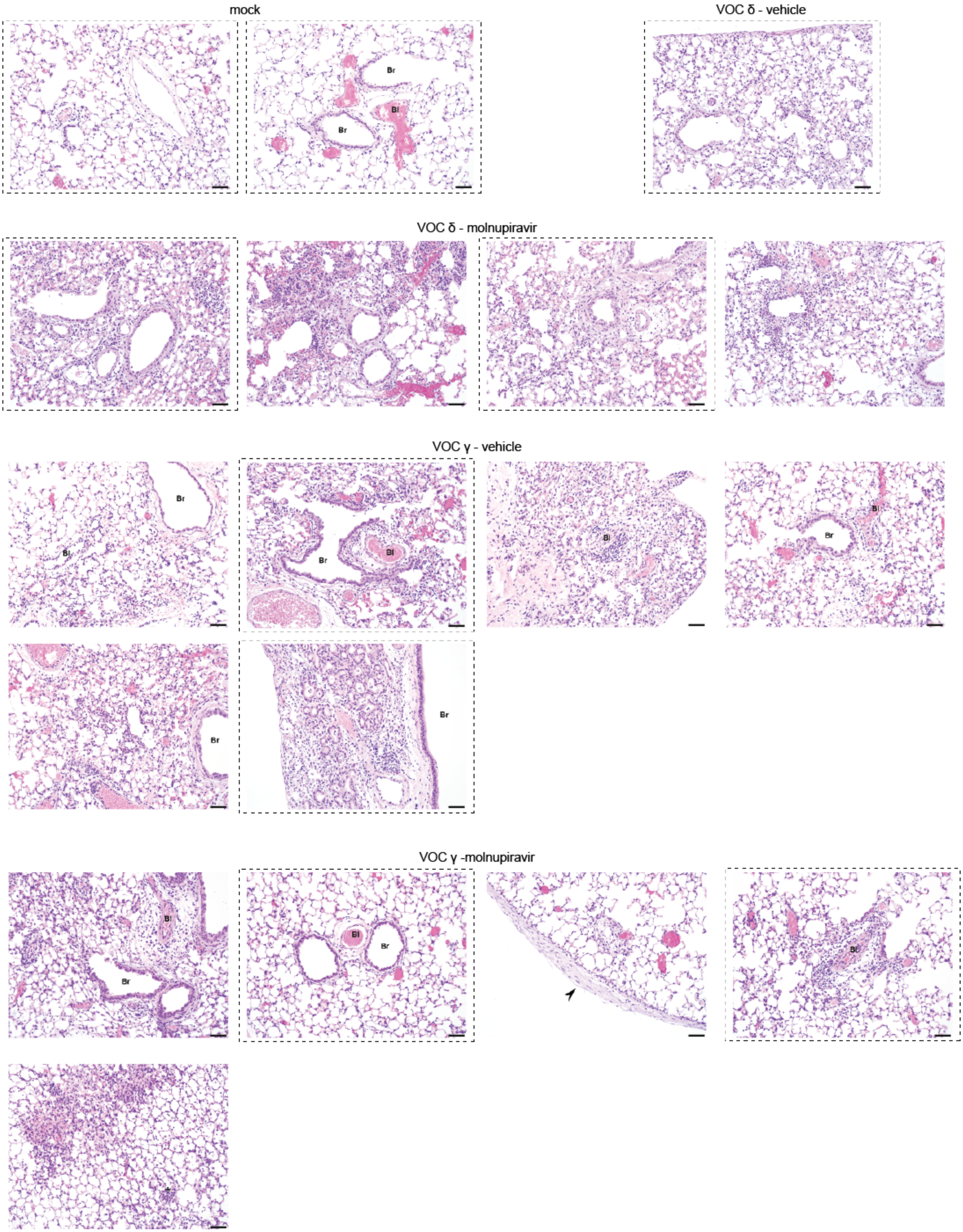
mock
VOC δ - vehicle
VOC δ - molnupiravir
VOC γ - vehicle
VOC γ -molnupiravir

8

Supplementary Fig. 14. Histopathology of dwarf hamster lungs 3 days after infection. Staining with hematoxylin and eosin. Histopathology photo-micrographs boxed out (dashed boxes) are shown in Fig. 6c. Each image represents tissue from an individual animal. Arrows highlight pleuritis. Br, bronchioles; Bl, blood vessel; scale bar 50 µm.

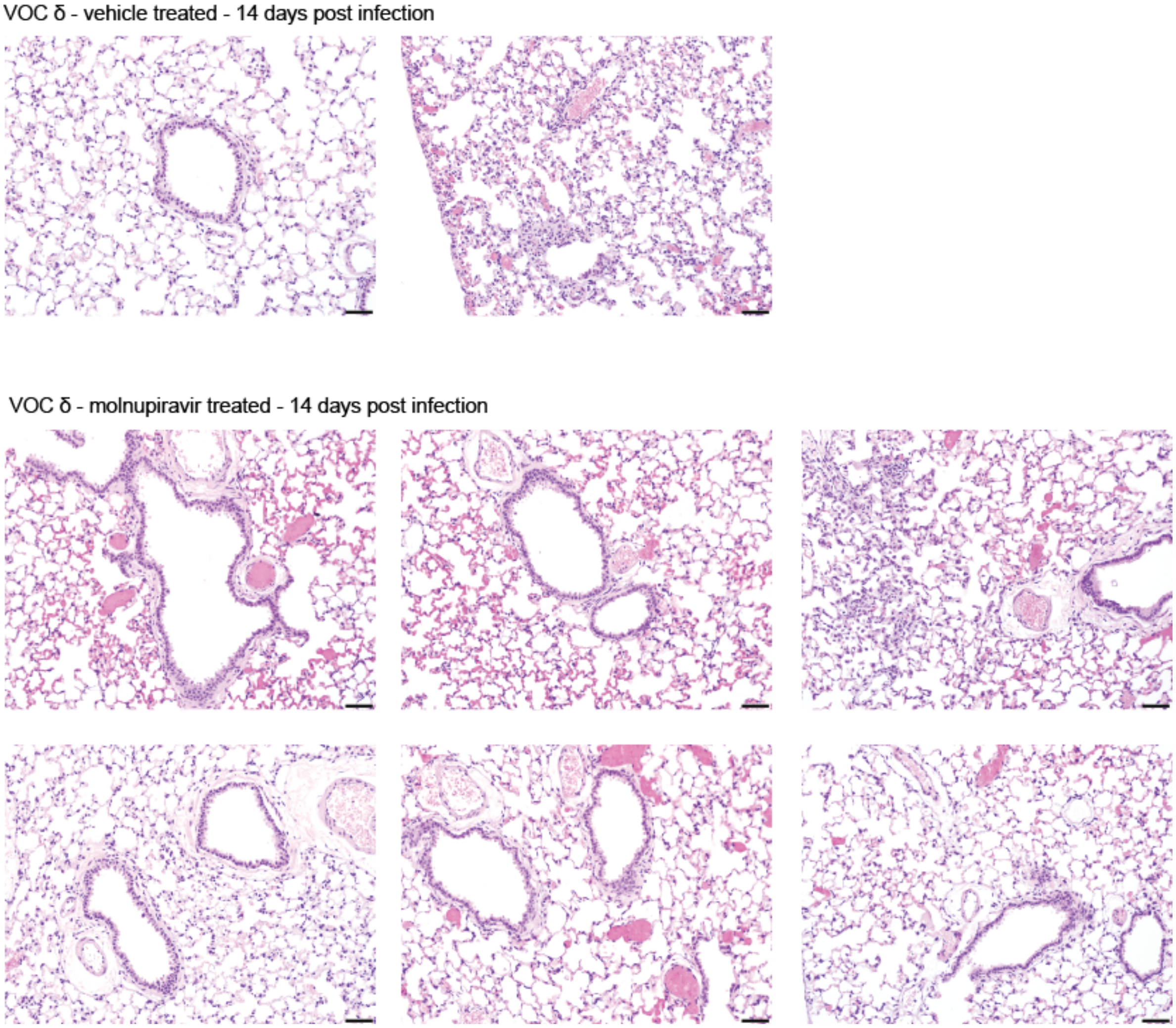

Supplementary Fig. 15. Histopathology of dwarf hamster lungs 14 days after infection. Staining with hematoxylin and eosin. Each image represents tissue from an individual animal. Scale bar 50 µm.
