# Supplementary material for: SARS-CoV-2 VOC type and biological sex affect molnupiravir efficacy in severe COVID-19 dwarf hamster model
Source: Nat Commun. 2022 Jul 29;13:4416. doi: 10.1038/s41467-022-32045-1 (PMC9338273; doi:10.1038/s41467-022-32045-1)
Supplement: Supplementary file 3 — Description of additional supplementary files [file 41467_2022_32045_MOESM3_ESM.pdf]

Description of additional supplementary files

Supplementary Dataset 1 : Complete statistical analyses of quantitative raw data .
