# Supplementary material for: SARS-CoV-2 VOC type and biological sex affect molnupiravir efficacy in severe COVID-19 dwarf hamster model
Source: Nat Commun. 2022 Jul 29;13:4416. doi: 10.1038/s41467-022-32045-1 (PMC9338273; doi:10.1038/s41467-022-32045-1)
Supplement: Supplementary file 5 — Reporting Summary [file 41467_2022_32045_MOESM5_ESM.pdf]

nature portfolio

Corresponding author(s): Richard K Plemper

Last updated by author(s): Jun 25, 2022

Reporting Summary

Nature Portfolio wishes to improve the reproducibility of the work that we publish. This form provides structure for consistency and transparency in reporting. For further information on Nature Portfolio policies, see our Editorial Policies and the Editorial Policy Checklist.

Statistics

For all statistical analyses, confirm that the following items are present in the figure legend, table legend, main text, or Methods section.

n/a	Confirmed	
☐	☒	The exact sample size (*n*) for each experimental group/condition, given as a discrete number and unit of measurement
☐	☒	A statement on whether measurements were taken from distinct samples or whether the same sample was measured repeatedly
☐	☒	The statistical test(s) used AND whether they are one- or two-sided<br>*Only common tests should be described solely by name; describe more complex techniques in the Methods section.*
☒	☐	A description of all covariates tested
☐	☒	A description of any assumptions or corrections, such as tests of normality and adjustment for multiple comparisons
☐	☒	A full description of the statistical parameters including central tendency (e.g. means) or other basic estimates (e.g. regression coefficient) AND variation (e.g. standard deviation) or associated estimates of uncertainty (e.g. confidence intervals)
☐	☒	For null hypothesis testing, the test statistic (e.g. *F*, *t*, *r*) with confidence intervals, effect sizes, degrees of freedom and *P* value noted<br>*Give P values as exact values whenever suitable.*
☒	☐	For Bayesian analysis, information on the choice of priors and Markov chain Monte Carlo settings
☒	☐	For hierarchical and complex designs, identification of the appropriate level for tests and full reporting of outcomes
☒	☐	Estimates of effect sizes (e.g. Cohen's *d*, Pearson's *r*), indicating how they were calculated

Our web collection on statistics for biologists contains articles on many of the points above.

Software and code

Policy information about availability of computer code

Data collection	Excel (Versions 16.42 and 16.43; Microsoft) and Numbers (version 10.1; Apple) used for most data collection. Reverse transcription qPCR data was collected using an Applied Biosystems StepOnePlus PCR system using the StepOnePlus software (Version 2.1; Applied Biosystems). Confocal microscopy images were captured using the Zeiss Zen 3.1 Blue software package (Windows 10).
Data analysis	Statistical analyses were performed in the Prism (GraphPad) software package (version 9.1.0). Figures were composed using Adobe Illustrator (version CS6). Confocal images were processed using Zeiss Zen Blue 3.. PK models were generated with Phoenix WinNonLin 8.3.3.33 (Certara) software package. Reverse transcription qPCR data was analyzed using the StepOnePlus software (Version 2.1; Applied Biosystems). SARS-CoV-2 next-generation sequencing analysis was performed using cutadapt (v1.9), bwa (version 0.7.17), Picard (version 2.18.15), VarScan (version 2.3), Annovar (2018Apr16 version), and Longitudinal Analysis of Viral Alleles (LAVA), available at (https://github.com/michellejlin/lava). Four-parameter variable slope regression modeling and statistical analyses were performed in the Prism (GraphPad) software package (Version 9.1.0). Power analyses were carried out using GPower 3.1 (University of Duesseldorf).

For manuscripts utilizing custom algorithms or software that are central to the research but not yet described in published literature, software must be made available to editors and reviewers. We strongly encourage code deposition in a community repository (e.g. GitHub). See the Nature Portfolio guidelines for submitting code & software for further information.

Data

Policy information about availability of data

All manuscripts must include a data availability statement. This statement should provide the following information, where applicable:

- Accession codes, unique identifiers, or web links for publicly available datasets
- A description of any restrictions on data availability
- For clinical datasets or third party data, please ensure that the statement adheres to our policy

The metagenomic sequencing reads generated in this study have been deposited in the NCBI BioProject database under accession code PRJNA803552 (https://www.ncbi.nlm.nih.gov/bioproject/?term=PRJNA803552). All other data generated in this study are provided in this published article and the Supplementary Information/Source Data file.

Field-specific reporting

Please select the one below that is the best fit for your research. If you are not sure, read the appropriate sections before making your selection.

☒ Life sciences ☐ Behavioural & social sciences ☐ Ecological, evolutionary & environmental sciences

For a reference copy of the document with all sections, see nature.com/documents/nr-reporting-summary-flat.pdf

Life sciences study design

All studies must disclose on these points even when the disclosure is negative.

Sample size	Appropriate sample sizes were determined using the Resource Equation and Power analysis. Unless otherwise noted, at least three samples were used for each group.
Data exclusions	No data was excluded from this manuscript.
Replication	All experimental data were reliably produced; the number of independent biological repeats and, when applicable, technical repeats is specified for each experiment in the figure legends. All experiments yielding quantitative raw data were independently repeated at least 3 times.
Randomization	Animals and samples were randomly sorted into experimental groups.
Blinding	Histopathology samples were blinded before analysis by a veterinary pathologist. For all other experiments, the investigators were not blinded to group allocation for data collection and analysis for any experiment performed in this study due to size of the research group with clearance for experimentation under high biocontainment conditions required for work with live SARS-CoV-2. Ferrets and dwarf hamsters cannot be handled by a single investigator under high biocontainment and resources available did not allow involvement of additional personnel that would have been required for additional blinding.

Reporting for specific materials, systems and methods

We require information from authors about some types of materials, experimental systems and methods used in many studies. Here, indicate whether each material, system or method listed is relevant to your study. If you are not sure if a list item applies to your research, read the appropriate section before selecting a response.

Materials & experimental systems

n/a	Involved in the study	
☐	☒	Antibodies
☐	☒	Eukaryotic cell lines
☒	☐	Palaeontology and archaeology
☐	☒	Animals and other organisms
☒	☐	Human research participants
☒	☐	Clinical data
☒	☐	Dual use research of concern

Methods

n/a	Involved in the study	
☒	☐	ChIP-seq
☒	☐	Flow cytometry
☒	☐	MRI-based neuroimaging

Antibodies

Antibodies used	Primary antibodies:<br>rabbit anti-SARS-CoV-2 Nucleocapsid monoclonal antibody (HL453) (Invitrogen, cat# MA5-36272)<br>mouse anti-SARS-CoV-1 and 2 Spike protein clone (1A9) monoclonal (Abcam, cat# ab273433)<br>mouse anti-MUC5AC (45M1) (ThermoFisher, cat# MA5-12175)

	rabbit anti-beta IV tubulin recombinant antibody conjugated with Alexa Fluor® 647 (EPR16775) (Abcam, cat# ab204034)<br>Conjugates:<br>donkey anti-goat (Alexa Fluor® 568 (ThermoFisher Scientific, cat# A-11057)<br>rabbit anti-mouse IgG (H+L) cross-adsorbed secondary antibody (Alexa Fluor® 488 (ThermoFisher Scientific, cat# A-11059)<br>goat anti-mouse IgG (H+L) highly cross-adsorbed secondary antibody (Alexa Fluor® 488 (Invitrogen, cat# A-11029)
Validation	Species and application validations for primary antibodies can be found in the antibody datasheets available at the following locations:<br>anti-SARS-CoV-2 Nucleocapsid (HL453) (https://www.thermofisher.com/antibody/product/SARS-CoV-2-Nucleocapsid-Antibody-clone-HL453-Monoclonal/MA5-36272)<br>anti-SARS-CoV-1 and 2 Spike protein (1A9) (https://www.abcam.com/sars-spike-glycoprotein-antibody-1a9-ab273433.html)<br>anti-MUC5AC (45M1) (https://www.thermofisher.com/antibody/product/Donkey-anti-Goat-IgG-H-L-Cross-Adsorbed-Secondary-Antibody-Polyclonal/A-11057)<br>anti-beta IV tubulin conjugated with Alexa Fluor® 647 (EPR16775) (https://www.abcam.com/alexa-fluor-647-beta-iv-tubulin-antibody-epr16775-ab204034.html)

Eukaryotic cell lines

Policy information about cell lines

Cell line source(s)	African green monkey kidney cells VeroE6 (ATCC®, cat# CRL-1586™)<br>African green monkey kidney cells VeroE6 stably expressing TMPRSS2 (VeroE6-TMPRSS2) (BPS Bioscience #78081)<br>human lung adenocarcinoma epithelial cells Calu-3 (ATCC® HTB-55™)<br>Human Bronchial Tracheal Epithelial cells (HBTEC) were derived from donor "M4", a 38-year old male (Lonza Bioscience, cat# CC-2540S, lot# 0000646466)
Authentication	Cells were authenticated by the supplier or by morphological appearance and susceptibility to virus infection including SARS-CoV-2.
Mycoplasma contamination	Cell lines were confirmed mycoplasma-negative when obtained from the supplier, followed by preparation and cryo-preservation of master and working stocks. Individual working stocks were replaced every three months. All cell lines in use in the laboratory were routinely retested for mycoplasma contamination in 6-months intervals and tested negative.
Commonly misidentified lines<br>(See ICLAC register)	No commonly misidentified cell lines were used in this study.

Animals and other organisms

Policy information about studies involving animals; ARRIVE guidelines recommended for reporting animal research

Laboratory animals	This study used female ferrets (Mustela putorius furo), family mustelids, genus mustela, 6-10 months of age and male and female Roborovski dwarf hamsters (Phodopus roborovskii), family cricetidae, genus phodopus, 3-10 months of age.
Wild animals	This study did not involve wild animals.
Field-collected samples	This study did not involve field-collected samples.
Ethics oversight	Experiments with SARS-CoV-2 involving ferrets were approved by the Georgia State Institutional Animal Care and Use Committee under protocol A20031. Experiments with SARS-CoV-2 involving Roborovski dwarf hamsters were approved by the Georgia State Institutional Animal Care and Use Committee under protocol A21019. All experiments using infectious SARS-CoV-2 were approved by the Georgia State Institutional Biosafety Committee under protocol B20016 and performed in BSL-3/ABSL-3 facilities at the Georgia State University.

Note that full information on the approval of the study protocol must also be provided in the manuscript.
